# Modeling Postnatal Hearing Case Finding Within the Italian National Health System

**DOI:** 10.3389/fped.2021.564662

**Published:** 2021-03-12

**Authors:** Eva Orzan, Giulia Pizzamiglio, Raffaella Marchi, Enrico Muzzi, Lorenzo Monasta, Lucio Torelli, Agnese Feresin

**Affiliations:** ^1^Institute for Maternal and Child Health Istituto di Ricovero e Cura a Carattere Scientifico (IRCCS) “Burlo Garofolo”, Trieste, Italy; ^2^Department of Medicine, Surgery and Health Sciences, University of Trieste, Trieste, Italy

**Keywords:** otitis media with effusion, family pediatrician, hearing impairment, newborn hearing screening, hearing surveillance

## Abstract

**Objective:** Despite the successful implementation of newborn hearing screening (NHS), a debate is emerging as to what should be the best means of enabling timely diagnosis and intervention for preschoolers with educationally significant sensorineural or conductive hearing impairment (HI) missed at the time of NHS or occurred after birth. Our study aims to document the proportion and characteristics of HIs diagnosed in children in need of audiologic assessment, in order to outline the optimization areas of an operational framework for auditory surveillance during preschool age.

**Method:** The referral routes and outcomes of 730 audiological assessments performed in 3 years within the framework of the early hearing identification program in Trieste (Italy) were retrospectively analyzed.

**Results:** Among 570/595 completed evaluations, an HI was diagnosed in 114 children, 73.7% of which presenting an exclusively conductive HI due to middle ear effusion. HIs were found in 36/141 who failed NHS, and 60/385 preschoolers who were referred by the primary care pediatrician's surveillance activity during well-child visits, with diagnostic yield of 25.5 and 15.5%, respectively.

**Conclusion:** Ongoing preschool surveillance in primary care setting integrated into a NHS program is feasible to conduct and may effectively identify HIs that missed NHS or were related with a risk factor. New triage instruments and protocols for immediate audiology referral could allow to obtain the diagnosis of educationally significant conductive and sensorineural HIs ahead of the development concern and in the same way reduce patient mobility, thus optimizing timing efficiency and economic impact of the program.

## Introduction

Auditory deprivation that follows undetected hearing impairments (HI) is responsible of delays in language competence, academic achievements and social and emotional development (1). These consequences are well-known for sensorineural and permanent HIs (2). However, even a conductive HI associated with a protracted otitis media with effusion (OME) may impair speech and language input for young children at their most sensitive age for language acquisition. A decrease in hearing sensitivity involves failure to respond appropriately to normal conversational speech or environmental sounds, behavioral changes, and problems with school performance and eventually with negative impact on the child's speech development (3).

Early intervention has demonstrated measurable benefit yet requires attaining a prompt access to the audiological evaluation in case of suspected HI. Even though newborn hearing screening (NHS) has proved to be an effective means to reducing the identification age of congenital HI, delays and loss to follow-up after failed NHS are common (4).

Moreover, concerns have been expressed regarding HIs that are not identified by the NHS (5) and a debate is growing on which should be the best means of facilitating a timely diagnosis and intervention for infants with delayed onset HI, as well as the conductive or sensorineural failures missed at the time of NHS (6).

With the aim of outlining the optimization areas of an operational framework for auditory surveillance during preschool age, we retrospectively analyzed the outcomes and diagnostic yield of the comprehensive audiological assessments performed at as part of the early hearing identification program in Trieste area, Italy. The program is characterized by an integration of screening levels [i.e., hospital-based objective screening and postnatal risk-screening offered by the family pediatrician (FP) during well-child visits] with the diagnostic audiologic level throughout preschool age.

## Methods

All children referred to the Pediatric Audiology Service (PAS) over three sample years (2013, 2015, and 2017) for a full audiological assessment were included in the study. The PAS, located at the “Burlo Garofolo” Institute in Trieste (Italy), is the referral center for three hospital-based NHSs and for the postnatal hearing surveillance run by roughly 60 FPs working in the territory. The annual live births in the population studied were 2,386 in 2013, 2276 in 2015, and 2276 in 2017. The regional program for the early identification of childhood HI, which includes the universal NHS and the hearing surveillance activity during preschool age, has been established since April 2012 (7).

Neonates are screened by nurses with a two-stage procedure: the first transient otoacoustic emission (TEOAE) screening is followed by automated auditory brainstem response (A-ABR) for infants who fail TEOAE; both tests are performed in at-risk infants. The NHS coverage was 95.7% in 2013, 96.8% in 2015, and 97.2% in 2017 with a referral rate of 3.9, 2.8, and 2.0%.

The FP, granted by the National Health System and deployed throughout the territory, is entrusted with the task of postnatal hearing surveillance performed at the regular health checks carried out at 1–3–6–9–12–18–36 months of age. Furthermore, the FP verifies the NHS completion, checks age-related auditory and language milestones, and monitors HI risk indicators.

Comprehensive audiological evaluations are provided at the PAS by trained audiologists, possibly in a single session. Objective and subjective evaluations include otoscopy, diagnostic TEOAE, tympanometry, air- and bone-conduction click-ABR for threshold measurement, and age-appropriate behavioral audiometry, when feasible.

Reasons for PAS admission were defined as follows: NHS failed testing for one or both ears (*NHS-fail*); missed or uncompleted NHS process (*NHS-miss*); passed NHS testing, but referred for congenital or postnatal risk-factor presence, as defined by the Joint Committee on Infant Hearing (JCIH) (1) (*NHS-pass with JCIH risk*); and passed NHS testing, but referred for other reasons, without presenting a risk factor as defined by JCIH (*NHS-pass without JCIH risk*).

The HI degree was defined in decibels (dB) hearing level (HL) as mild (>20– ≤ 40), moderate–severe (>41– ≤ 79), and profound (>80 HL) by averaging the hearing threshold at 500, 1,000, and 2,000 Hertz (Hz).

Results of audiometric tests performed at the PAS appointment were cross-checked and summed up in four auditory statuses: 1. *Normal hearing* (hearing threshold and middle ear functioning within the normal range, type A tympanogram); 2. *Conductive HI* due to middle-ear effusion (impaired air-conduction threshold, bone conduction within the normal range, non-evocable TOAE, type B or C tympanogram); 3. *Sensorineural-mixed HI* (impaired air- and bone-conduction threshold, non-evocable TEOAE, any type of tympanogram); and 4. *Not completed*. (i.e., hearing profile not obtained within 1 month from the start of the evaluation, because of partial or non-concluded testing, lack of compliance, instrumental problems, etc.).

Within the analyzed years, the PAS registered 730 requests for audiological evaluation, involving 595 children (371 boys and 224 girls) with an average age of 31.3 months (min: 0.36; max: 80.5 months).

In the case of multiple accesses of the same child, the hearing profile was defined only by the first completed PAS assessment, and the results of subsequent evaluations were excluded from the data analysis.

### Statistics

The comparisons of proportion were made using 2020 MedCalc Software Ltd. *P*-values < 0.05 were considered significant. Data were analyzed using Excel (version 16.23) for IOS 10.14.6.

## Results

The audiological assessments were brought to completion in 570/595 children referred to the PAS within the analyzed years. There was a significant reduction in the number of children who requested 2 or more evaluations from 2013 to 2017 (22.5 and 3.5% of evaluated children, respectively, *P* < 0.0001). Regarding the reasons for PAS admission, there is a decreasing trend in the proportion of children who failed or missed NHS, without however achieving statistical significance along the analyzed years. An HI was identified in 114 children, with mostly bilateral involvement (78.9%). In 73.7% of HI cases, the deficit resulted to be exclusively due to middle-ear effusion (i.e., conductive type) of mild or moderate degree, with a large majority (79.7%) presenting only a mild threshold involvement. The sensorineural-mixed HI represented 26.3% of the identified HIs (5 mild, 24 moderate–severe, and 1 with a profound degree of involvement). In 10/30 children, the sensorineural-mixed HI was unilateral.

The proportion of identified conductive HIs appreciably (although not significantly) increased from 2013 to 2017, in parallel with sensorineural HI decreasing proportion. Table 1 summarizes the obtained results.

**Table 1:** The table summarizes the collected data.

**Year of analysis**	**a.** **Total *N* of evaluations**	**b.** **Total *N* of evaluated children**	**c.** ***N* of children requiring two or more evaluations (% of b.)**	**d.** **Reasons for referral of evaluated children (% of b.)**				**e.** ***N* of children with completed evaluation (% of b.)**	**f.** ***N* of children with identified HI (% of e.)**	**g.** **HI type (% of f.)**	
				**NHS fail**	**NHS miss**	**NHS pass + JCIH risk**	**NHS pass without JCIH risk**			***N* of children with conductive HI**	***N* of children with Sensorineural or mixed HI**
2013	309	240	54 (22.5)	59 (24.6)	34 (14.1)	125 (52.1)	22 (9.2)	224 (93.3)	53 (23.6)	32 (60.4)	21 (39.6)
2015	272	211	48 (22.7)	58 (27.5)	20 (9.5)	109 (51.7)	24 (11.4)	207 (98.1)	32 (15.5)	24 (75.0)	8 (25.0)
2017	149	144	5 (3.5)	24 (16.6)	15 (10.4)	88 (61.1)	17 (11.8)	139 (96.5)	29 (20.9)	28 (96.6)	1 (3.4)
Total	730	595	107 (17.9)	141 (23.7)	69 (11.6)	322 (54.1)	63 (10.6)	570 (95.8)	114 (20.0)	84 (73.7)	30 (26.3)
		*P*-value^*^	*P* < 0.0001	*P* = 0.0655	*P* = NS	*P* = 0.0862	*P* = NS	*P* = 0.1836	*P* = NS	*P* = 0.0004	*P* = 0.0004

The panels on the left include the analyzed years and the total number of requested evaluations and evaluated children. The fourth column indicates also the number of children who requested two or more evaluations. The central panel resumes the admission motives divided into the four referral profiles: NHS fail, NHS miss, NHS miss with JCIH risk, and NHS pass without JCIH risk (see materials and methods for clarification). The panel on the right side shows the outcomes of the completed audiologic evaluations, indicating the number of children with diagnosed HI and the type of HI.

* The comparison of proportions was calculated between years 2013 and 2017. P value is considered significant if <0.05. P = NS not significant.

*N, number; NHS, neonatal hearing screening; HI, hearing impairment; JCIH, Joint Committee on Infant Hearing*.

Overall, the presence of at least one JCIH risk factor (*NHS pass with risk*) was the most frequent reason for PAS admission, attributed to 56.5% of children that were fully evaluated (322/570), with a yield of 48 (15%) cases with conductive and 4 (1.3%) with sensorineural/mixed HI. The single most reported risk factor was “caregiver concern regarding hearing, speech, language, or developmental delay” (169/595), which represented the referral reason of 39 hearing impaired children, 35 of which presented a conductive HI. Other reported risk factors included hospitalization in neonatal intensive care unit for more than 5 days and/or use of ototoxic medications (127/595); family history of permanent childhood HI (72/595); *in utero* infections, such as CMV (27/595); craniofacial anomalies (21/595); neurodegenerative disorders or syndromes associated with HI (18/595); postnatal infections associated with sensorineural hearing loss (11/595); head trauma (2/595); and chemotherapy (2/595).

*NHS fail* and *NHS miss* access profiles displayed the major yield of sensorineural/mixed HIs, 20/139 (14.4%) and 5/63 (7.9%), respectively.

**Figure 1:**
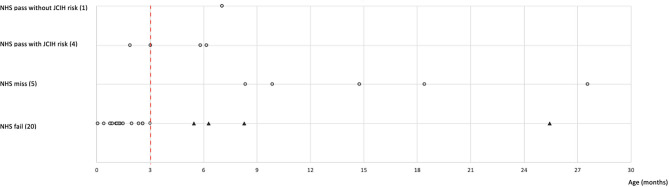
The graph highlights the age of completed audiological evaluation in children with sensorineural or mixed HI (gray dots) according to reasons of referral. The number of children for each reason of referral is indicated in brackets. Some dots overlap because the patients underwent the visit in the same period of time. The black triangles indicate NHS fail children who completed the PAS assessment beyond the recommended 3 months of age (red dashed line). NHS, neonatal hearing screening; JCIH, Joint Committee on Infant Hearing; HI, hearing impairment; PAS, pediatric audiology service.

The ages at which the sensorineural/mixed HI was identified were segregated by reason of access (Figure 1). Within *NHS fail* referrals, 16/20 (80%) of sensorineural/mixed HIs resulted within the JCIH benchmark of 3 months of life for HI diagnosis. Differently, the mean age of sensorineural/mixed HI identification for *NHS miss* referrals (5 cases) was 15.8 months (min 8.3, max 27.6).

## Discussion

Ongoing surveillance of infants and toddlers in primary care settings is feasible to conduct and can effectively overcome the barriers to follow-up in childhood hearing deficits described by other reports (6, 8). NHS and postneonatal routes of hearing surveillance represent two sides of the same theme in preventive hearing care, and current childhood hearing screening programs should today include not only newborns but also infants and children after the newborn period, so as to intercept the HIs missed at NHS and those that arise after the neonatal age (1). In spite of the accomplished awareness on the great importance of avoiding unidentified infants' and toddlers' HI, there is a paucity of research addressing access to follow-up services for children identified after the newborn period (6). Our study proves that in a territory where birthing centers, primary care, and audiologic services establish direct collaborative relationships and work together to facilitate access to audiological evaluation, the overall capacity and efficiency of the HI identification program may take relevant advantage. In the scenario analyzed, the sustainability of these interventions is based both on the duties of the FPs, whose health surveillance activity is regulated by the National Health System, and on the availability of dedicated PASs, with equipment and qualified medical and technical personnel, as regulated from the specific regional program. Despite the uniqueness of the Italian FP-based system of primary care on the European and global scene, our study, even though descriptive, may well represent an example of program strategies for minimizing loss to follow-up and form a basis for comparison for other childhood hearing prevention programs.

There are some interesting remarks gathered from the present data that could become useful in the phases following the implementation of an integrated service. Firstly, the number of referrals and assessed children has halved over the years in our program, offering diverse interpretations. The variation cannot be explained solely by the drop in the birth rate, which decreased by not more than 6.8% from 2013 to 2017 in the area (9). A more extensive contribution to the decrease in referrals may be linked to an initial work overload for the PAS: the percentage of children who requested 2 or more evaluations to obtain a definitive hearing profile significantly decreased after 3 years of activity, possibly representing a “learning curve” of the PAS operators in optimizing evaluation times. A further explanation could finally arise from the distribution of the referrals' reasons along the analyzed years, and in particular for those referrals relating to the NHS: as expected, in the years following the introduction of our program (2012), we appreciated both a decrease in the proportion of newborns failing NHS testing and, accordingly, also a decrease of referrals of children that missed NHS (7).

As a second observation, 82.7% (115/139) of *NHS fail* referrals were evaluated by the PAS within 3 months of age in our program. The percentage reaches 87.8% if we included 8 of the 25 late evaluated infants who had a history of prematurity that were nevertheless evaluated within 3 months of corrected age. Although overall PAS coverage for *NHS fail* cases does not reach the benchmarks indicated by JCIH (1), the outcomes of this program demonstrate greater timing efficiency compared to other reports (8, 10). Regarding other motives of referral, it is worth recalling that, as part of the Italian National Health System, the FP's office is a common door through which almost all children pass for health visits, giving the opportunity to reach most of the children at risk and most of those missed by the NHS during their preschool age (11). Even though significance is not always achieved in our data comparison to confirm this supposition, some uncertainties may become clinically significant with greater sample size.

A third comment regards the fact that positive audiological evaluations are overloaded by conductive HIs linked to middle-ear effusions (OME). An HI related to OME can be a major concern as it may impair speech and language input for young children at their most sensitive age for language acquisition (3). The overall prevalence of OME rate ranges from 4 to 20% in different regions of the world and varies according to age: about 50% of cases occur in infants under the age of 1, and 60% occur in infants under the age of 2 (12). The condition is frequently asymptomatic, and the associated conductive HI of mild or moderate degree often resolves spontaneously, yet requires a follow-up to confirm HI resolution or a prompt consultancy for tube surgery in case of prolonged HI (13) or in children with sensory, physical, cognitive, or behavioral factors that place them at increased risk for speech or developmental comorbidities (14). Similarly to literature (15), OME HI accounted for 44% of HI diagnoses linked to NHS failures and 87% of postnatal referrals in our population.

The reported numbers should alert pediatric health organizations not to overload the PASs with assessments that could be managed and resolved already in primary care, without interfering with the workload of second-level audiological services. It is the belief of the authors that an effective means to increasing the power of suspected-oriented level of audiological referral would be favored by providing pediatric primary care with effective self-help hearing screening objective equipment. An ongoing program during preschool age could in fact result in additional public health benefits: it would allow a reduction of family mobility in accessing to more expensive audiologic evaluations. Moreover, a self-help diagnosis would favor a more aware and appropriate prescription of antibiotic therapy in case of otitis media (16), possibly contributing to the strategies of the national plan against antimicrobial resistance. Ultimately, a better triage of children with HI would allow a more targeted referral of OMEs that require a surgical decision, with further reduction in direct and indirect surveillance costs. Self-help audiometric equipment should be designed specifically for mild-to-moderate HI level detection and should discriminate HI type, in order not to miss prompt identification or sensorineural or mixed HIs (17). There have been some attempts in this regard which would deserve further investigation. For example, Eiserman et al. (18) proposed an OAE screening using a multistep protocol to help early childhood health providers screen children for permanent HI (18). Recently, Bhatia et al. (19) reported on OAE technology coupled with tympanometry to allow physicians to better triage patients for immediate audiology referral (19).

As for the limits of this work, our data are descriptive and based on PAS outcomes without follow-up of children who did not present a risk indicator, i.e., not all children born and living in the area have been re-screened for hearing at a later age. The yield of the program may have been consequently overestimated.

Regarding permanent (i.e., sensorineural) HI, Watkin and Baldwin conducted a cohort study over a 10-year period in Great Britain, after initiation of NHS (20). They reported that 51% of children entering primary school were identified with permanent HI after the neonatal period. Mean age at identification of postnatal permanent HI was 3.8 years, and case finding in childhood after the NHS was through care pathways reactive to professional or parental concern (20). In our series, 10/30 (33.3%) of sensorineural-mixed HIs were identified outside the context of NHS, proving a greater postneonatal HI detection.

In the near future, further research should be addressed to confirm the strength of an ongoing hearing prevention service during preschool age. Studies should be designed to include the use of an objective hearing screening tool to be implemented already at primary care level. In addition to defining the organizational and economic sustainability of the system, data analyses should also be set up with a view and aim of promoting the quality of primary care provided as well as economic and time saving for public health organizations.

## Data Availability Statement

The raw data supporting the conclusions of this article will be made available by the authors, without undue reservation.

## Ethics Statement

The studies involving human participants were reviewed and approved by the institutional review board (research project 17/17) and by the Italian Ministry of Health-CCM (project 12/2013). Written informed consent to participate in this study was provided by the participants' legal guardian/next of kin.

## Author Contributions

EO and AF: conceived and designed the experiment. AF, GP, and RM: collected the data. LT, GP, and LM: analyzed the data. EO: wrote the paper. LT and EM: provided critical review of the paper. All authors contributed to the article and approved the submitted version.

## Conflict of Interest

The authors declare that the research was conducted in the absence of any commercial or financial relationships that could be construed as a potential conflict of interest.

## Abbreviations

NHS: newborn hearing screening
HI: hearing impairment
OME: otitis media with effusion
TEOAE: transient oto-acoustic emission
OAE: oto-acoustic emission
A-ABR: automated auditory brainstem response
PAS: pediatric audiology service
FP: family pediatrician
JCIH: Joint Committee on Infant Hearing
HL: hearing level.
